# Subspecies hybridization as a potential conservation tool in species reintroductions

**DOI:** 10.1111/eva.13191

**Published:** 2021-02-08

**Authors:** Lilith J. Zecherle, Hazel J. Nichols, Shirli Bar‐David, Richard P. Brown, Helen Hipperson, Gavin J. Horsburgh, Alan R. Templeton

**Affiliations:** ^1^ School of Biological and Environmental Sciences Liverpool John Moores University Liverpool UK; ^2^ Mitrani Department of Desert Ecology Jacob Blaustein Institutes for Desert Research Ben‐Gurion University of the Negev Midreshet Ben‐Gurion Israel; ^3^ NERC Biomolecular Analysis Facility Department of Animal and Plant Sciences University of Sheffield Sheffield UK; ^4^ Department of Bioscience Swansea University Swansea UK; ^5^ Department of Biology Washington University St. Louis MO USA

**Keywords:** conservation management, *Equus hemionus*, genetic admixture, reintroduction, subspecies hybridization

## Abstract

Reintroductions are a powerful tool for the recovery of endangered species. However, their long‐term success is strongly influenced by the genetic diversity of the reintroduced population. The chances of population persistence can be improved by enhancing the population's adaptive ability through the mixing of individuals from different sources. However, where source populations are too diverse the reintroduced population could also suffer from outbreeding depression or unsuccessful admixture due to behavioural or genetic barriers. For the reintroduction of Asiatic wild ass *Equus hemionus* ssp. in Israel, a breeding core was created from individuals of two different subspecies (*E. h. onager* & *E. h. kulan*). Today the population comprises approximately 300 individuals and displays no signs of outbreeding depression. The aim of this study was a population genomic evaluation of this conservation reintroduction protocol. We used maximum likelihood methods and genetic clustering analyses to investigate subspecies admixture and test for spatial autocorrelation based on subspecies ancestry. Further, we analysed heterozygosity and effective population sizes in the breeding core prior to release and the current wild population. We discovered high levels of subspecies admixture in the breeding core and wild population, consistent with a significant heterozygote excess in the breeding core. Furthermore, we found no signs of spatial autocorrelation associated with subspecies ancestry in the wild population. Inbreeding and variance effective population size estimates were low. Our results indicate no genetic or behavioural barriers to admixture between the subspecies and suggest that their hybridization has led to greater genetic diversity in the reintroduced population. The study provides rare empirical evidence of the successful application of subspecies hybridization in a reintroduction. It supports use of intraspecific hybridization as a tool to increase genetic diversity in conservation translocations.

## 1 INTRODUCTION

Reintroductions are an important and powerful tool in the conservation and recovery of endangered species. The long‐term goal of a viable self‐sustaining population is strongly dependent on the genetic makeup (Seddon & Armstrong, 2016), yet evolutionary genetic considerations are often neglected in applied conservation management (Mace & Purvis, 2008). Small numbers of founders and genetic bottlenecks experienced during the establishment phase cause an increased risk of inbreeding and reduction in genetic diversity due to random genetic drift (Frankham et al., 2002). Consequently, reintroduced populations often display low levels of genetic diversity and reduced evolutionary potential which can impact the populations' chances of long‐term persistence (Jamieson, 2010). The capture of sufficient genetic diversity is therefore critical for reintroduction success and should be prioritized when selecting founders (Seddon & Armstrong, 2016). A major practical difficulty is that for many endangered species, potential source populations are small and the number of individuals available for translocation is often limited. In these cases, it has been suggested that genetic diversity could be increased by mixing individuals from different source populations (Jahner et al., 2019; McLennan et al., 2020; Neuwald & Templeton, 2013). Indeed, Biebach and Keller (2012) compared 40 reintroduced populations of Alpine ibex (*Capra ibex*) and reported that the degree of admixture in founder individuals had a greater impact on heterozygosity in the established population than the number of founders.

The mixing of source populations prior to a reintroduction is not without risks, with the potential for outbreeding depression having been highlighted (Edmands, 2007; Huff et al., 2011). If founders stem from different geographical or ecological regions, individuals may have developed local adaptations. Population admixture is expected to break up these coadaptations, resulting in reduced fitness of the hybrid descendants, especially in later generations (Tallmon et al., 2004; Templeton et al., 1986). Additionally, genetic incompatibility or behavioural differences between the source populations may prevent successful interbreeding of founder individuals (Gottsberger & Mayer, 2019). Complete or partial admixture barriers have been reported between different subspecies and populations of the same species (Soland‐Reckeweg et al., 2009). For example, female brown boobies (*Sula leucogaster*) actively selected against males of a different color morph, thereby preventing hybridization of different genetic clusters within the same species (López‐Rull et al., 2016). A barrier to admixture could impact the success of a reintroduction by effectively creating two cryptic populations of smaller size, leading to increased extinction risk.

The application of intraspecific hybridization as a conservation management tool, while much debated, remains highly controversial (Allendorf et al., 2001). This is also due to a general lack of empirical studies on immediate and long‐term effects on the translocated populations. The reintroduction of the Asiatic wild ass in Israel provides a rare study system to investigate the impact of this potentially powerful yet controversial strategy on reintroduction outcome. In 1968, a captive breeding core was established with 11 individuals from two different subspecies, the Iranian onager (*Equus hemionus onager*) and the Turkmen kulan (*E. h. kulan*) (Saltz et al., 2000). Breeding was unmanaged, and individuals of the different subspecies were allowed to interbreed (Saltz & Rubenstein, 1995). Between 1982 and 1987, 28 individuals (14 females, 14 males) and between 1992 and 1993 an additional 10 individuals (7F, 3M) were released into the Negev desert. The reintroduced population has since rapidly increased in size and expanded its range across a large geographical area in Southern Israel (Gueta et al., 2014). No signs of outbreeding depression in the population have been observed. In fact, the reproductive rate of the population is higher than in other reintroduced Asiatic wild ass populations, the reproductive success of females is high, and the reintroduction is currently considered a success (Renan et al., 2018; Saltz & Rubenstein, 1995). Nonetheless, a major question is whether this success followed admixture of the two subspecies (potentially increasing genetic diversity) or whether genetic or behavioural barriers led to distinct cryptic populations. Differences in habitat selection between parental subspecies or between hybrids and parental subspecies could prevent complete admixture. Indeed, a previous analysis of subspecies‐specific mitochondrial haplotypes indicated spatial structuring of the population (Gueta et al., 2014).

The aim of this study was to investigate the long‐term genetic consequences of sourcing founders from two different subspecies in a conservation reintroduction. We first explored levels of admixture in the historic captive breeding core and in the current wild population. Then, we investigated population genomic parameters including effective populations size estimates and heterozygosity levels, to evaluate the genetic status of the populations before and after release.

## 2 MATERIALS AND METHODS

### 2.1 DNA sample collection

In 1991, three generations after the establishment of the captive population, whole blood samples were collected from 25 individuals in the breeding core (hereafter *founder population*; Gueta et al., 2014). Between 2011 and 2017, a total of 33 blood and tissue samples of the reintroduced population (hereafter *wild population*) were collected opportunistically during veterinary treatments, fitting of radio collars and from animals killed in traffic accidents from across the population's range (Figure 2; Table S1). In addition, we obtained tissue and whole blood samples of the *E. h. onager* (*N* = 6) and *E. h. kulan* (*N* = 15) subspecies from captive populations in European zoos, collected opportunistically during veterinary treatments or from dead individuals (Table S1). Whole blood samples were stored in EDTA tubes (BD Vacutainer K2EDTA 18.0 mg; Thermo Fisher Scientific; Vacuette K3EDTA 3 mg, Greiner Bio‐One), and tissue samples were stored either untreated in paper bags or in screw‐cap tubes in 70% ethanol. All samples were stored frozen (at −20°C or −80°C).

### 2.2 DNA extraction, ddRADseq library preparation and sequencing

We used commercial silica spin column‐based extraction kits (Qiagen DNeasy Blood and Tissue Kit, Thermo Fisher Scientific GeneJET Genomic DNA Purification Kit) to purify DNA, following the manufacturers protocol. Samples were digested with the high‐fidelity versions of the restriction enzymes *Eco*RI (R3101S; New England Biolabs) and *Sbf*l1 (R3642L; NEB). The ddRADseq library preparation followed a protocol adapted from Peterson et al. (2012). Libraries were pooled in equimolar amounts (100 nM) and sequenced on one lane of an Illumina HiSeq4000 flowcell. Sequencing produced over 400 million raw paired end reads with a mean read length of 300 bp. We assessed the quality of raw reads with the FastQC tool (Andrews, 2010) and a mean Phred+33 quality score >30 was recorded for all bases. Reads were de‐multiplexed, and barcodes and Illumina adapters were trimmed using the *process_radtags* script in the Stacks 2.0 pipeline (Catchen et al., 2013). We used seven replicate samples and a parameter optimization approach adapted from Paris et al. (2017) to assemble loci de novo (optimal parameters: ‐m3 ‐N0 ‐M4 ‐n4). Subsequent alignment of de novo assembled loci to a reference genome resulted in significant reduction of assembled loci (Table S2). Since mean individual coverage was exceptionally high (114×) and SNP error rates calculated from seven replicate pairs of samples were low for de novo assembly (mean ± SD = 0.011 ± 0.003; Table S2), we decided to continue with the de novo assembled loci. Called SNPs were filtered in VCFtools (minimum mean individual coverage ≥35×, minimum minor allele count ≥3, SNPs present in minimum of 80% of individuals, Hardy–Weinberg equilibrium outliers for *α* = 0.05; Danecek et al., 2011). Eight samples were removed due to low sequencing quality. The final data set contained 69 unique individuals and 4231 SNPs.

### 2.3 Admixture of the subspecies

To investigate levels of admixture in the Israeli populations, we calculated hybrid indices for each individual. First, we analysed the zoo samples (sample sizes: kulan *n* = 9, onagers *n* = 5) to identify diagnostic SNPs, which are fixed for alternative alleles in the two subspecies and calculated hybrid indices as the proportion of alleles inherited from either subspecies (M. Kardos, pers. comm.). Out of the total of 4231 SNP positions, two were not detected in any of the onagers and were removed from the analysis. Of the remaining 4229 SNPs, only 13 (0.3%) were found to be fixed for different alleles in the two subspecies populations.

We calculated individual hybrid indices both from diagnostic SNPs and using maximum likelihood (ML) methods implemented in the R package *introgress* (Gompert & Buerkle, 2010) on the entire data set. *introgress* provides estimates of parental allele frequencies and calculates hybrid indices, accounting for uncertainty in allele ancestry when parental populations share alleles. The zoo onager and kulan samples were specified as parental populations with allele ancestry information being used to calculate individual hybrid indices (as the proportion of kulan ancestry) for other samples. We further investigated admixture levels using pairwise fixation indices (*F*_ST_) and calculated for all pairs of populations in the *hierfstat* R package (Goudet, 2005). We tested Weir and Cockerham's pairwise *F*_ST_ values for significant deviation from 0 using the function *boot*.*ppfst* and 10,000 bootstrap permutations. Additionally, we investigated individual admixture levels using the Bayesian cluster algorithm implemented in the program Structure (Pritchard et al., 2000). To investigate the admixture between two ancestral populations, we ran Structure with the admixture model and correlated allele frequencies, and we predefined the number of genetic clusters (*K*) as 2. We set the alternative ancestry prior (POPALPHAS = 1) which allows for distinct *α*‐values for each ancestral population, as recommended when sampling is unbalanced (Wang, 2017). Runs were performed with 1 × 10^6^ iterations of the Markov Chain Monte Carlo (MCMC) chain preceded by 1 × 10^5^ burn‐in iterations and 10 repetitions.

We further investigated the relationship between the sampled populations using multivariate methods in the *adegenet* R package (Jombart, 2008). We first used a principal component analysis (PCA) to explore the data set without a priori groupings. We then performed a discriminant function analysis of the first 15 principal components (referred to as DAPC), which accounted for 52% of the total variance. The DAPC fits orthogonal discriminant functions that maximize between group relative to within‐group variation for a priori defined groups (Jombart et al., 2010). We defined four groups consistent with the sampled populations.

To test for a potential barrier to admixture caused by different habitat preferences between the subspecies, we investigated spatial autocorrelation between individuals of the wild population based on hybrid indices in ArcGIS 10.0 (ESRI, 2011). We set a fixed distance threshold of 3400 m between sample locations with row standardization to ensure that each individual had at least one neighbour.

### 2.4 Heterozygosity, inbreeding and effective population sizes

To avoid any bias due to obvious linkage disequilibrium between SNPs, we used a reduced data set with only 1 SNP per locus (total *N* = 1738) for this analysis. We calculated expected (*H*_e_) and observed heterozygosity (*H*_o_) and the mean inbreeding coefficient across loci for each population using the *pegas* R package (Paradis et al., 2010). We expressed individual heterozygosity as the proportion of heterozygote markers for each individual. We estimated variance (*N*_ev_) and inbreeding (*N*_ef_) effective population sizes using the program NeEstimator V2 (Do et al., 2014). *N*_ev_ refers to the size of an ideal population which displays the same sampling variance in allele frequencies as the focal population (Nei & Tajima, 1981). We estimated *N*_ev_ using the temporal method (Waples, 1989) with the founder and the wild population used as two samples of the same population at generation 0 and generation 3 (based on generation time of 7.5 years, Ransom et al., 2016). Standardized variance in allele frequencies were computed using the method described by Pollak (1983). *N*_ef_ describes the size of an ideal population with the same probability of alleles being identical by decent as the population in question. We used the linkage disequilibrium method to estimate *N*_ef_ for the founder and the wild population (Waples & Do, 2008). For both estimates of *N*_e_, we set the lowest allele frequency to 0.02 to avoid bias in estimates and we calculated jackknifed 95% confidence intervals (CIs), as recommend for larger numbers of markers (Do et al., 2014).

## 3 RESULTS

### 3.1 Admixture of the subspecies

The mean population hybrid indices differed significantly between estimates based on 13 diagnostic SNPs (Mean = 0.521, SD = 0.148) and those obtained by maximum likelihood including all 4229 SNPs (Mean = 0.475, SD = 0.054; Paired Student's *t*‐test: *t*_54_ = −2.732, *p* < 0.01; Figure S1). Nonetheless, these differences had only a minor impact on the biological interpretation of the results, as both methods indicated high admixture levels. Since the identified diagnostic SNPs were few with unknown locations in the genome, they could be clumped and poorly represent genome‐wide admixture levels. Therefore, we used hybrid indices estimated from maximum likelihood methods in subsequent analyses. The founder and the wild population displayed high levels of individual admixture (Figure S2). There was a significant difference in the mean (±SD) hybrid indices between the founder (0.502 ± 0.061) and the wild populations (0.453 ± 0.037; unequal variances *t*‐test: *t*_37.84_ = −3.543, *p* < 0.001). These values indicated a very even subspecies ancestry in the founder population which shifted after release to a slightly higher proportion of onager ancestry in the wild population. This was consistent with pairwise *F*_ST_ values, which were all significant at *p* < 0.05. The founder and wild population both displayed a stronger genetic differentiation from the kulans than from the onagers (Table 1A).

**TABLE 1:** Population genetic differentiation (A) and diversity indices (B) for captive and admixed reintroduced populations of *Equus hemionus* ssp

(A)	*F*_ST_ ^a^			
	Onager	Kulan	Founder	Wild
Onager	−	*	*	*
Kulan	0.149	−	*	*
Founder	0.049	0.149	−	*
Wild	0.179	0.248	0.154	−

B)	*H*_e_ ^b^	*H*_o_ ^c^	*F*_IT_ ^d^
Onager	0.201	0.226**	−0.117
Kulan	0.223	0.251**	−0.116
Founder	0.261	0.289**	−0.093
Wild	0.253	0.258**	−0.019**

^a^ Weir & Cockerham's pairwise *F*_ST_ value

^b^ Expected heterozygosity.

^c^ Observed heterozygosity.

^d^ Mean individual inbreeding coefficient.

* Significance level (*p* < 0.05) for the pairwise *F*_ST_ values (10,000 bootstrap permutations).

** Significance level (*p* < 0.001) for the paired *t*‐test comparison of *H*_e_ and *H*_o_ for all populations and for independent *t*‐test comparison of *F*_IT_ between founder and wild population.

**FIGURE 1:**
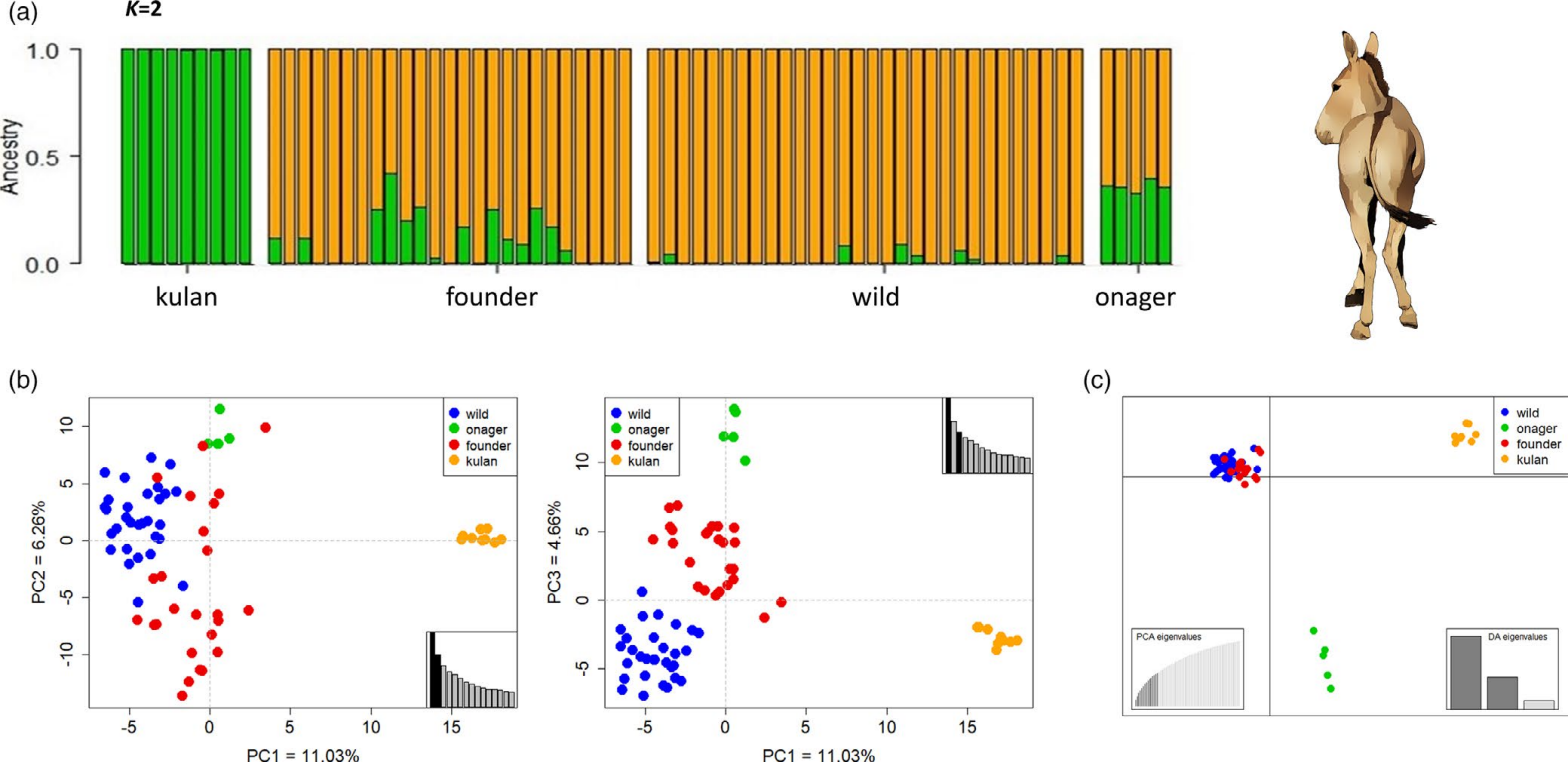
Subspecies admixture analysis. (a) Structure bar plot for *K* = *2*. Each bar represents an individual, and sampled populations are separated by black lines. The colours indicate admixture of inferred ancestral clusters. (b) Principal component analysis (PCA) based on the first 15 principal components (PCs) (retaining 52.44% of the total variance). PC1 (11.03%) separates kulans, while the other populations are differentiated by PC3 (4.66%). Insets display eigenvalues of PCs. (c) Discriminant analysis of PCs displaying founders and wild populations clustering closely together, separated from kulans and onagers along the *x*‐ (DA1) and *y*‐axes (DA2), respectively. The analysis was based on the first 15 PCs which explain 52.40% of the total variance and three discriminant axes (DA) retaining all of this variance (DA1 = 62.45%, DA2 = 29.47%, DA3 = 8.08%). Top right inset displays eigenvalues of PCs of the PCA, and top left inset displays eigenvalues of the retained DAs of the DAPC

**FIGURE 2:**
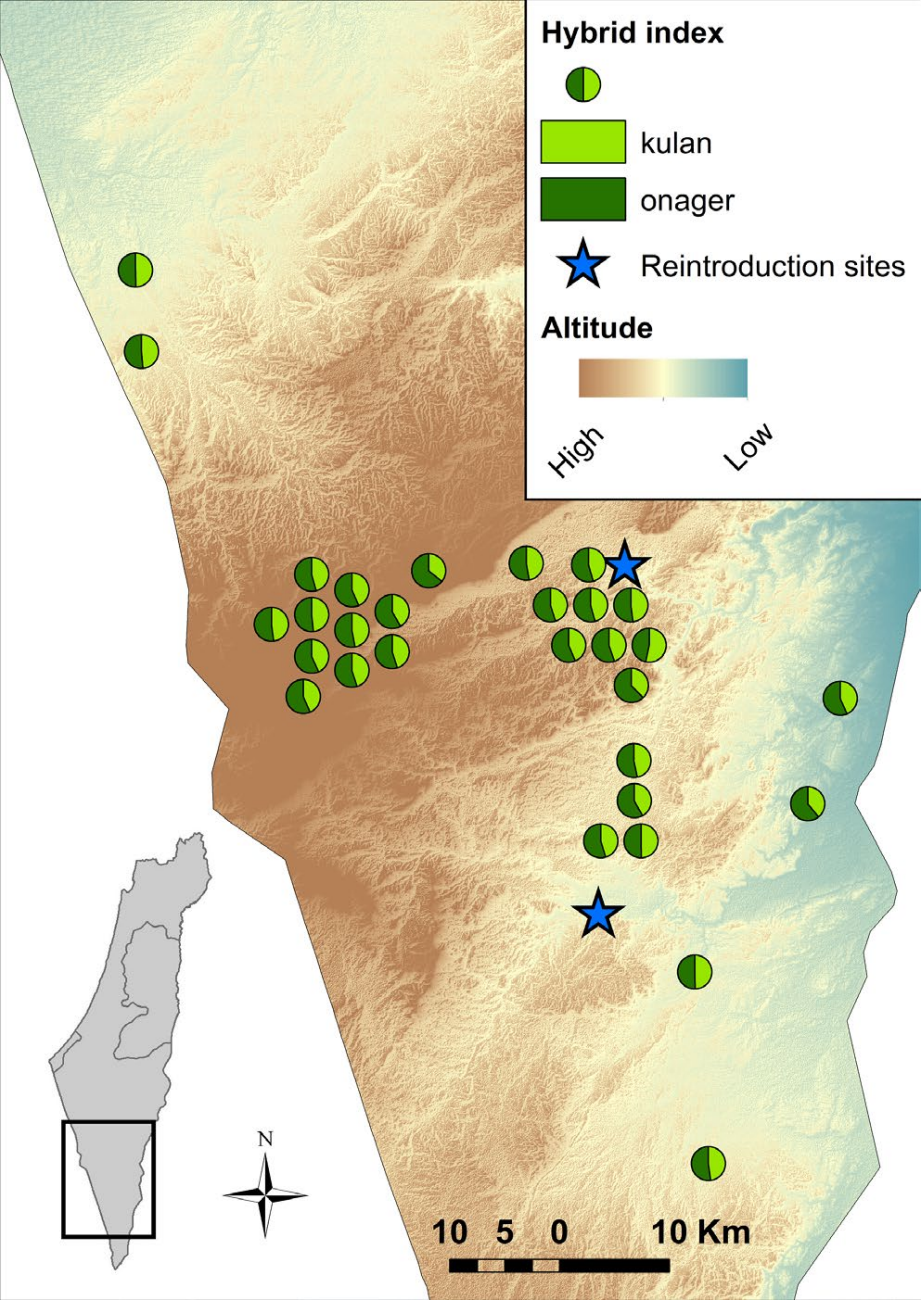
Spatial autocorrelation. Spatial distribution of individual samples from the wild population. Pie charts represent sampling location and maximum likelihood estimates of individual hybrid indices based on 4229 SNPs

In the Bayesian clustering approach, the two inferred ancestral populations did not coincide with the subspecies samples. While kulans were clearly differentiated from the other populations, onagers displayed greater levels of admixture, comparable to the Israeli populations (Figure 1a). This is consistent with the results of the multivariate analyses. The PCA clearly separated the kulans from the other samples along PC1 (11.03% of total variance), while there was some overlap between the onagers, founder and wild populations along PC2 (6.26%; Figure 1b). This overlap was resolved along PC3 (4.66%) which differentiated the data into four clusters, consistent with the original populations (Figure 1b). In the DAPC, founders and the wild population, which clustered closely together, were clearly separated from both subspecies. The first discriminant axis which explained most of the variation (DA1 = 62.45%) separated the hybrid population from the kulans, while the second DA (29.47%) differentiated between the hybrids and the onagers (Figure 1c). Taken together, the results of the admixture analysis display high levels of subspecies admixture with a slight bias towards increased onager ancestry in the wild population. The spatial analysis revealed no signs of spatial autocorrelation between individuals based on hybrid indices (Moran's *I* = −0.045, *z* = −0.367, *p* = 0.713, Figure 2), suggesting association between habitat preference and subspecies ancestry.

### 3.2 Heterozygosity, inbreeding and effective population sizes

The observed heterozygosity was significantly higher than expected in all population (Table 1B). *H*_o_ was the highest in the founder population and significantly greater than in the zoo populations of onagers (unequal variances *t*‐test: *t*_3307_ = −7.852, *p* < 0.001) and kulans (*t*_3411.1_ = −4.944, *p* < 0.001; Table 1B). Furthermore, comparison of the Israeli founder and wild population showed a 10.85% loss of heterozygosity during the reintroduction. This difference was found to be significant (*t*_3452.5_ = 4.586, *p* < 0.001). The founder population also displayed significantly greater variation in individual heterozygosity levels (range = 0.157, SD = 0.037) than the wild population (range = 0.111, SD = 0.024) (Bartlett's test: *K*‐squared(1) = 5.194, *p* < 0.05; Figure S3). The population‐level inbreeding coefficient *F*_IT_ was low in all populations, yet significantly smaller in the founder population than in the wild (independent samples *t*‐test: *t*_2867.4_ = −11.201, *p* < 0.001; Table 1B).

Effective population size estimates for the wild and the founder population were small. For the wild population, the temporal method estimated a variance effective population size (95% CIs) of *N*_ev_ = 17.1 (15.8–18.6), while the linkage disequilibrium method estimated an inbreeding effective size of *N*_ef_ = 27.6 (19.2–43.9). For the founder population, the estimated inbreeding effective size was even smaller at *N*_ef_ = 7.1 (3.2–12.6).

## 4 DISCUSSION

The reintroduced Asiatic wild ass population in Israel was founded with individuals from two different subspecies, *E. h. onager* and *E. h. kulan*. Our population genomic analysis demonstrated high levels of admixture between these subspecies in the breeding core (founder population), after three generations of unmanaged mating. Admixture levels remained high in the reintroduced wild population, yet with a slight bias towards increased onager ancestry. No spatial autocorrelation arising from subspecies ancestry was detected in the wild population. These results indicated that there were no genetic or behavioural barriers to subspecies hybridization and that no cryptic subpopulations have formed due to a lack of admixture. This may be due to weak genetic differentiation between the subspecies; we found that only 0.03% of the analysed SNPs were fixed for opposite alleles in the onager and kulan samples, which is consistent with previous findings demonstrating only low genetic divergence between onagers and kulans (Bennett et al., 2017). Species such as the Asiatic wild ass, which show low levels of divergence between subpopulations or subspecies may therefore present good candidates for hybridization as part of genetic management.

Further supporting the potential benefits of hybridization as a conservation tool, we discovered that the hybrid founder population showed significantly higher observed heterozygosity than the zoo onager and kulan populations. There was a 10.85% reduction in observed heterozygosity from the founder to the wild population, although this was still above levels found in the zoo onager and kulan populations and was significantly greater than the expected heterozygosity for the wild population. A loss of heterozygosity is commonly observed in reintroduced populations and likely the result of a genetic bottleneck due to a subset of just 38 individuals from the founder population being released into the wild (Broders et al., 1999; Grossen et al., 2018; Mock et al., 2004). This could be further intensified by strong genetic drift due to extreme polygyny in the species (Greenbaum et al., 2018; Renan et al., 2018). Similarly, reintroduced populations often display very small effective population sizes as found in the Israeli wild ass population (Manlick et al., 2018; Murphy et al., 2018). However, if the effective population sizes remain small over an extended period, this can seriously threaten the populations' long‐term persistence (Franklin, 1980). Continued genetic monitoring of the populations will be necessary to adjust management, should the effective population sizes not increase. Other studies have also highlighted the need for continued management of small and isolated reintroduced populations to avoid long‐term genetic erosion (Jamieson, 2010; Saremi et al., 2019; Vonholdt et al., 2008).

The zoo populations of kulan and onager also showed significant heterozygote excess. This could be due to managed breeding by zoos as both subspecies are part of the European Endangered Species Programs of the European Association of Zoos and Aquaria, which aims to maximize retained genetic diversity in the captive populations (EAZA, 2019). Heterozygote excess could also be enhanced by population substructure, which was previously reported for the captive onager population, whereby heterozygosity could be enhanced if individuals from different clusters were interbred (Nielsen et al., 2007). Stochastic effects causing random changes in allele frequencies between the sexes can also result in an observed heterozygote excess, commonly observed in small populations (Templeton, 2018).

We found that the Bayesian clustering algorithm and the multivariate analyses clearly distinguished kulans but not onagers from the Israeli founder and wild populations. The results are consistent with the hybrid indices and pairwise *F*_ST_ values which identified a stronger differentiation between both Israeli populations and kulans. The observed pattern likely relates to the true founders of the Israeli population. In this study, we used samples collected from zoo populations to represent the parental subspecies. However, due to the small population size and drift likely experienced by the captive populations, the sampled individuals might not accurately represent the original individuals that established the breeding core. If the kulans sampled in this study are genetically differentiated from the kulans that were brought to Israel, this could explain the greater similarity between the onagers and the hybrid population. Finally, accidental hybridization of the captive onager population could also explain the observed increased admixture levels in onagers. While we cannot rule out that accidental hybridization may have occurred in captivity, there are no indications for hybridization in the specimen records of samples used in this study. Furthermore, captive populations of kulans and onagers are strictly separated and no hybrids have been recorded since 2008 (Pohle, 2014).

Overall, this study provides valuable insights that are useful for the management of remaining onager and kulan populations. Both subspecies are currently classified as endangered by the IUCN and a major conservation concern is the extreme fragmentation and small sizes of remaining wild populations (Bennett et al., 2017; Kaczensky et al., 2018). Furthermore, on the basis of numerous phylogenetic analyses (Geigl & Grange, 2012; Orlando et al., 2009; Vilstrup et al., 2013), some authors advocate a revision of the separate subspecies status of *E. h. onager* and *E. h. kulan* and joint management of the remaining populations (Bennett et al., 2017; Oakenfull et al., 2000). The results presented here demonstrate that genetic or behavioural barriers are unlikely to compromise a mixed stock management approach in in situ and ex situ conservation.

Our analysis has wider implications for conservation translocations in general. The reintroduction in Israel is a rare success in *E. hemionus* conservation. To date, 18 attempts have been recorded, yet only five reintroductions (including the one in Israel) succeeded in establishing large free‐ranging populations (>100 individuals and stable or increasing population trend, Kaczensky et al., 2016). The admixed Israeli population displayed fast postrelease population growth, with female net reproductive rate (*R*_0_ = 1.87) exceeding those observed in other reintroduced populations of *E. hemionus* (Saltz & Rubenstein, 1995). Similarly, White et al. (2018) reported faster postrelease population growth in admixed Australian mammal populations compared to populations of single source origin. These results suggest that admixture may directly improve reintroduction success by enhancing critical postrelease population growth. In fact, there is increasing evidence that mixing of different genetic lineages can improve the chances of successful population establishment and persistence by increasing the population's genetic diversity and adaptive potential (Biebach & Keller, 2012; Mueller et al., 2020; Tordoff & Redig, 2001). In the case of *E. hemionus* ssp. in Israel, controlled experiments would be necessary to verify whether the hybridization was critical to this rare reintroduction success, and these are not currently feasible. Nevertheless, our results are in‐line with a growing body of literature which suggests that sourcing founder individuals from different subspecies, or genetically differentiated populations, can enhance the population genetic diversity.

Despite increasing evidence that intraspecific hybridization holds enormous potential for species recovery programmes, deliberate admixture of individuals from different subspecies or even genetic lineages remains highly controversial. This is because of the associated risk of outbreeding depression if source populations are genetically too divergent (Frankham et al., 2011). The resulting reduced reproductive fitness and loss of local adaptations is often cited as the reason to avoid mixing as it could jeopardize successful population establishment (Edmands, 2007; Templeton et al., 1986). Additionally, there may be concerns of compromising the genetic uniqueness of rare populations. While the risk of outbreeding depression is real and deserves careful consideration, it has likely been overstated in many cases (Frankham, 2015), whereas inbreeding depression is a more imminent threat to many small and isolated populations (Frankham et al., 2011). Moreover, genetic differentiation between populations due to recent isolation and genetic drift must not be mistaken for local adaptations (Templeton, 1996). Weeks et al. (2016) warned against conservation strategies aimed at preserving genetic differences in recently fragmented populations, thereby intensifying their genetic erosion.

Intentional admixture may be considered as a conservation tool for systems where the risk of outbreeding depression is low. While still much research is needed on how to accurately predict the risk of outbreeding depression, a cautious approach could include a first stage of careful assessment of the genetic differentiation between potential source populations (e.g. see Frankham et al., 2011 for best practice guidelines). This may be followed by a second stage, where interbreeding and individual fitness of hybrid offspring are monitored in a captive breeding facility for at least two generations, as fitness effects may be obscured in the F1 generation (Edmands, 2007). If no fitness reductions are recorded, the final (third) stage involves release of admixed individuals into the wild.

While there is increasing evidence for the potential benefits of mixed source reintroductions, long‐term genetic data of successfully admixed reintroduced populations are scarce. Yet such case studies are important for evaluating this strategy as a conservation tool and advancing general conservation translocation guidelines. Our study therefore represents an advance through provision of valuable empirical data on a successful and complete admixture between individuals from two different subspecies.

## CONFLICT OF INTEREST

None declared.

## Supporting information

Supplementary Material

## Data Availability

The data that support the findings of this study are openly available in NCBI at https://www.ncbi.nlm.nih.gov/, SRA accession: PRJNA650264 (Zecherle et al., 2020).
