# Supplementary material for: Subspecies hybridization as a potential conservation tool in species reintroductions
Source: Evol Appl. 2021 Feb 8;14(5):1216–24. doi: 10.1111/eva.13191 (PMC8127701; doi:10.1111/eva.13191)
Supplement: Supplementary file 1 — Supplementary Material [file EVA-14-1216-s001.docx]

# Supplementary information

# Subspecies hybridization as a potential conservation tool in species reintroductions

L.J.Zecherle^1,2,4+^, l.j.zecherle@2016.ljmu.ac.uk, lillyzecherle@gmail.com

H.J.Nichols^3^, hazel h.j.nichols@swansea.ac.uk

S.Bar-David^2^, shirlibd@bgu.ac.il

R.P.Brown^1^, R.P.Brown@ljmu.ac.uk

H.Hipperson^4^, h.hipperson@sheffield.ac.uk

G.J. Horsburgh^4^, g.horsburgh@sheffield.ac.uk

A.R.Templeton^5^, temple_a@wustl.edu

^1^School of Biological and Environmental Sciences, Liverpool John Moores University, Byrom Street, Liverpool, L3 3AF, UK

^2^Mitrani Department of Desert Ecology, Jacob Blaustein Institutes for Desert Research, Ben-Gurion University of the Negev, Midreshet Ben-Gurion, 84990, Israel

^3^Department of Bioscience, Swansea University, Singleton Park, Swansea, SA2 8PP, UK

^4^NERC Biomolecular Analysis Facility, Department of Animal and Plant Sciences, University of Sheffield, Western Bank, Sheffield, S10 2TN, UK

^5^Department of Biology, Washington University, St. Louis, Missouri, 63110-1093, USA

+ Corresponding author: Email: l.j.zecherle@2016.ljmu.ac.uk, lillyzecherle@gmail.com; Tel: +49(0)1628490360

**Table S1** Information on DNA samples collected from the different study populations.

| Population | Subspecies | N^*^ | Sex | Sample Type | Source |
| --- | --- | --- | --- | --- | --- |
| Founder | *E.hemionus ssp.* | 25 (0) | 13F, 12M | Blood | HaiBar Yotvata Reserve, Israel |
| Wild | *E.hemionus ssp.* | 33 (3) | 11F, 15M,  7 unknown | Blood (N=13) and Tissue (N=20) | Negev desert, Israel |
| Onager | *E.h.onager* | 6 (1) | 5F, 1M | Blood | Chester Zoo, UK (N=4)  Wildlands Adventure Zoo, Emmen, Netherlands (N=2) |
| Kulan | *E.h.kulan* | 15 (4) | 5F, 6M,  4 unknown | Blood (N=11) and Tissue (N=4) | Nuremberg Zoo, Germany (N=11)  Rostock Zoo, Germany (N=4) |
| Replicates  (Founder, Wild) | *E.hemionus ssp.* | 7 (0) | 3F, 4M | Blood (N=4) and Tissue (N=3) | HaiBar Yotvata Reserve (N=2) and Negev desert (N=5), Israel |

^*^ Numbers in parenthesis indicate samples excluded due to poor sequencing quality

**Table S2** *De novo* assembly in Stacks pipeline (Catchen et al. 2013) and subsequent reference alignment to one of two reference genomes. Number of assembled loci, polymorphic loci and SNPs shared among at least 80% of individuals are compared. Mean ±SD SNP error rates for different methods are given.

| Parameters | *De novo* | *E. asius^[[1]](#footnote-1)^* | *E. caballus^[[2]](#footnote-2)^* |
| --- | --- | --- | --- |
| Assembled loci | 3135 | 1598 | 1182 |
| Polymorphic loci | 2639 | 1326 | 949 |
| SNPs | 5981 | 2867 | 1970 |
|  |  |  |  |
| SNP error rate^[[3]](#footnote-3)^ |  |  |  |
| Mean | 0.0108 | 0.0143 | 0.0144 |
| SD | 0.0031 | 0.0050 | 0.0052 |

**Fig. S1** Mean hybrid index, expressed as proportion of *E.h.kulan* ancestry, differed significantly between estimates based on 13 diagnostic SNPs (Mean=0.521, SD=0.148) and those obtained by maximum likelihood using all 4,229 SNPs (Mean=0.475, SD=0.054; Paired Student's t-test: *t*_(54)_=-2.732, p<0.01).

**
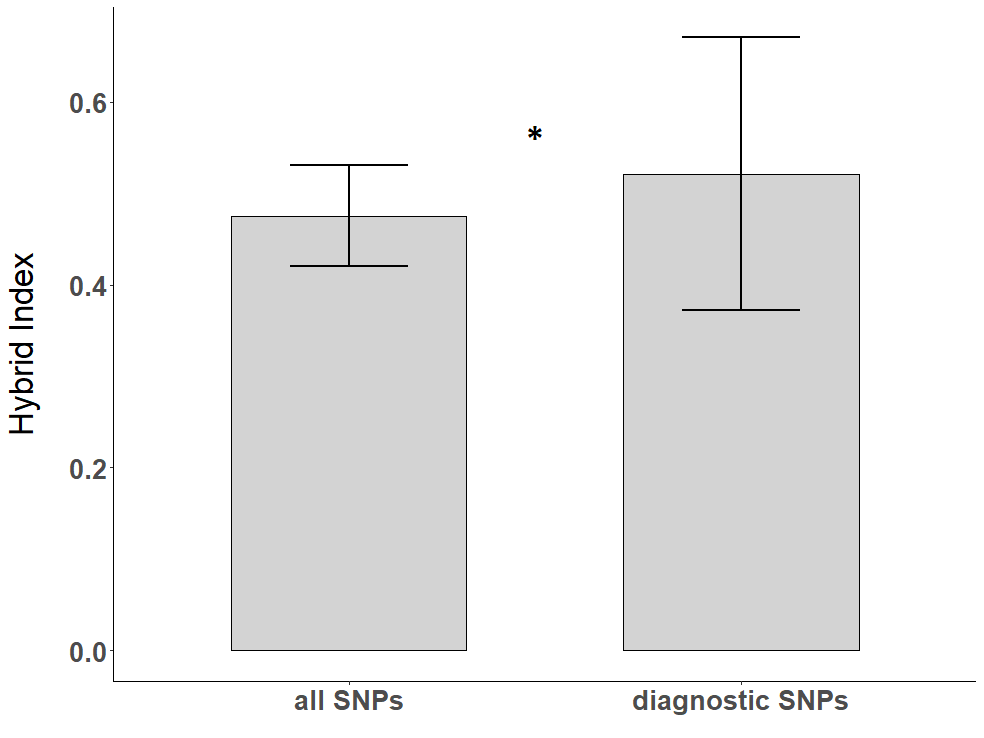
**

**Fig. S2** Subspecies admixture analysis. A) Admixture plots as produced by the *introgress* R package for the founder (left) and the wild (right) population. Horizontal bars indicate individuals, vertical lines indicate 4229 SNPs. Individual hybrid index as proportion kulan ancestry is indicated by line plots on the right.

**
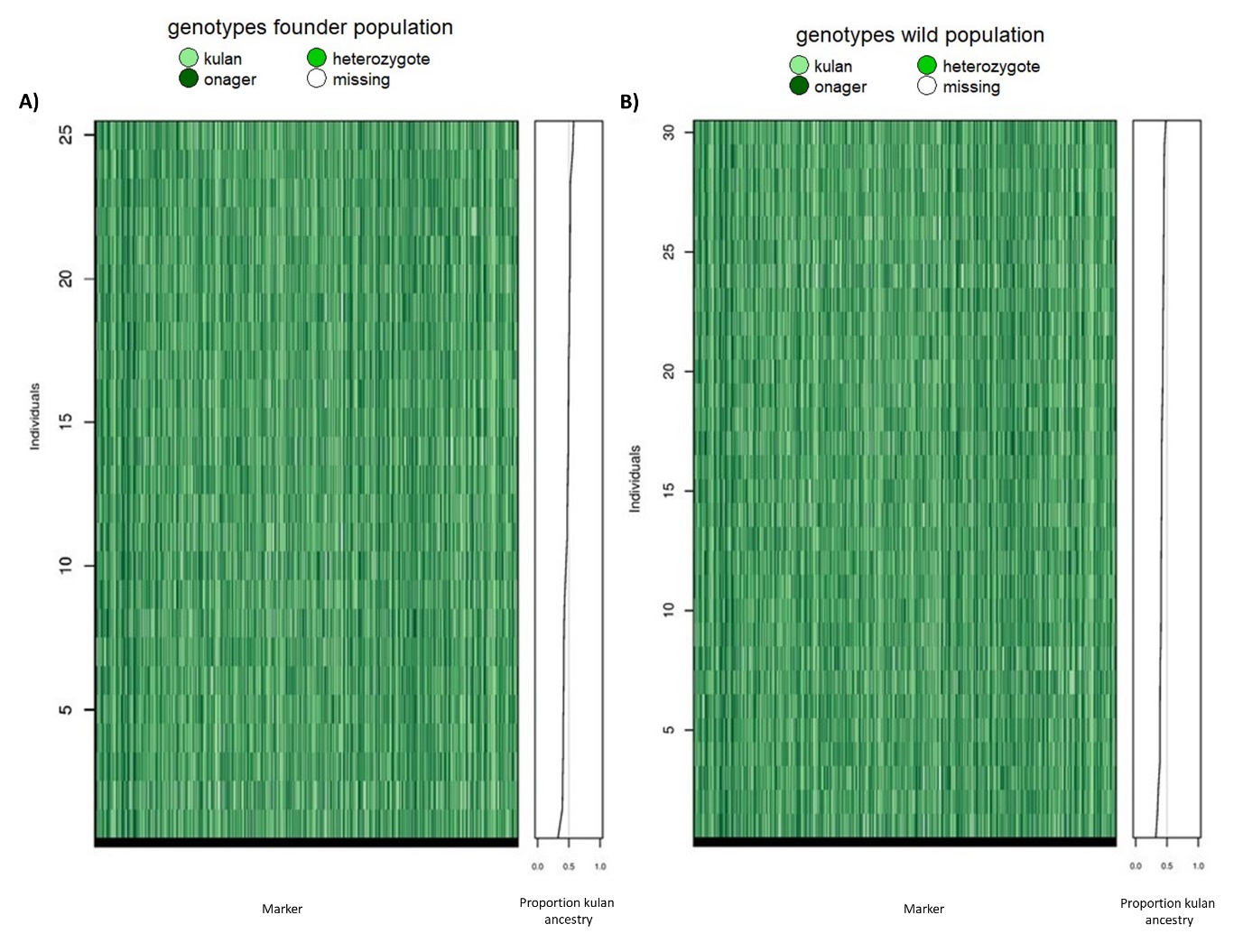
**

**Fig. S3** Variation in individual heterozygosity by population. Individual IDs are on the x-axis, proportion of heterozygote markers on the y-axis. Red lines indicate population means.

**
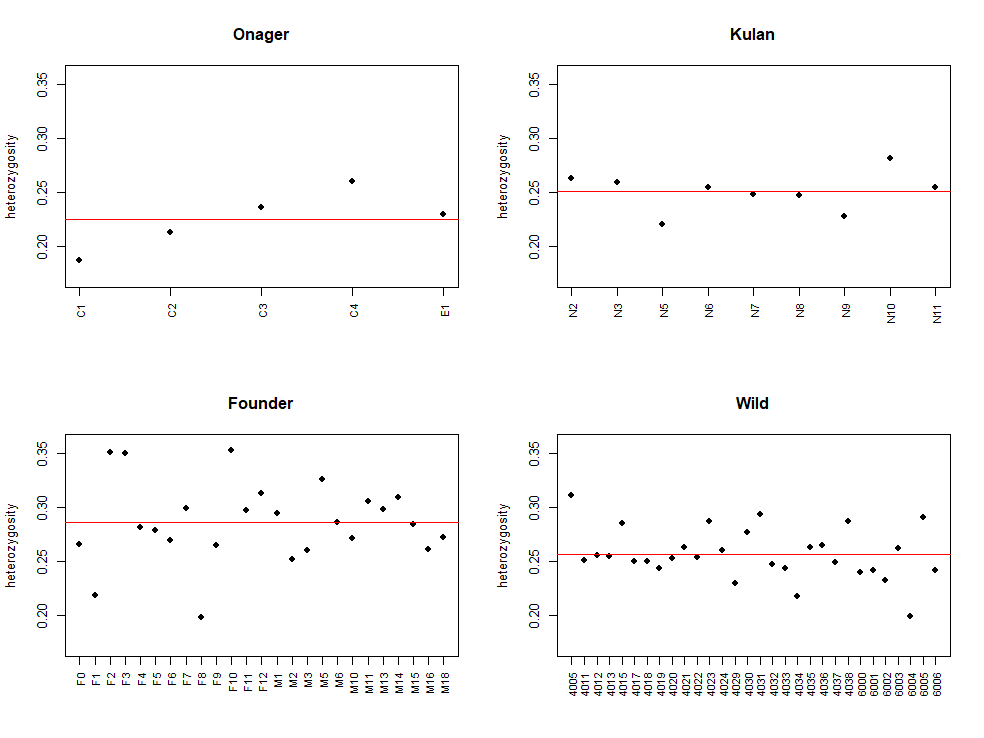
**

1. domestic donkey *Equus asinus*, NCBI accession GCF_001305755.1 [↑](#footnote-ref-1)
2. domestic horse *Equus caballus*, NCBI accession GCF_002863925.1 [↑](#footnote-ref-2)
3. number of SNP mismatches within a pair of replicate samples, divided by

   the total number of SNPs present in the individual. A total of 7 replicate pairs

   were included. [↑](#footnote-ref-3)
